# The Predictive but Not Prognostic Value of *MGMT* Promoter Methylation Status in Elderly Glioblastoma Patients: A Meta-Analysis

**DOI:** 10.1371/journal.pone.0085102

**Published:** 2014-01-13

**Authors:** An-an Yin, Lu-hua Zhang, Jin-xiang Cheng, Yu Dong, Bo-lin Liu, Ning Han, Xiang Zhang

**Affiliations:** 1 Department of Neurosurgery, Xijing Institute of Clinical Neuroscience, Xijing Hospital, Fourth Military Medical University, Xi’an, Shaanxi Province, The People's Republic of China; 2 Department of Prosthodontics, School of Stomatology, Fourth Military Medical University, Xi’an, Shaanxi Province, The People's Republic of China; Johns Hopkins Hospital, United States of America

## Abstract

**Background:**

The clinical implication of O6-methylguanine-DNA methyltransferase (MGMT) promoter status is ill-defined in elderly glioblastoma patients. Here we report a meta-analysis to seek valid evidence for its clinical relevance in this subpopulation.

**Methods:**

Literature were searched and reviewed in a systematic manner using the PubMed, EMBASE and Cochrane databases. Studies investigating the association between MGMT promoter status and survival data of elderly patients (≥65 years) were eligible for inclusion.

**Results:**

Totally 16 studies were identified, with 13 studies included in the final analyses. The aggregate proportion of MGMT promoter methylation in elderly patients was 47% (95% confidence interval [CI]: 42–52%), which was similar to the value for younger patients. The analyses showed differential effects of MGMT status on overall survival (OS) of elderly patients according to assigned treatments: methylated vs. unmethylated: (1) temozolomide (TMZ)-containing therapies: hazard ratio [HR] 0.49, 95% CI 0.41–0.58; (2) TMZ-free therapies: HR 0.97, 95% CI 0.77–1.21. More importantly, a useful predictive value was observed by an interaction analysis: TMZ-containing therapies vs. RT alone: (1) methylated tumors: HR 0.48, 95% CI 0.36–0.65; (2) unmethylated tumors: HR 1.14; 95% CI 0.90–1.44.

**Conclusion:**

The meta-analysis reports an age-independent presence of MGMT promoter methylation. More importantly, the study encouraged routine testing of MGMT promoter status especially in elderly glioblastoma patients by indicating a direct linkage between biomarker test and individual treatment decision. Future studies are needed to justify the mandatory testing in younger patients.

## Introduction

Glioblastoma is the most frequent brain malignancy and is invariably associated with very poor prognosis, despite the use of multiple treatment modalities including maximal tumor resection, radiotherapy (RT) and chemotherapy.[1] Glioblastoma becomes more common among elderly population in recent years, and the prognosis of older patients is even poorer with the typical median overall survival (OS) range of only 4–6 months. [2]–[4].

The standard treatment for elderly glioblastoma patients remains suboptimal.[2] RT is the current mainstay for most elderly patients.[2] Moreover, temozolomide (TMZ)-containing therapies including postoperative TMZ alone or in combination with RT are being widely evaluated in the clinical settings.[2] Given that elderly glioblastoma patients are heterogeneous subgroups with different prognostic variables and different responses to treatments, clinically relevant factors are needed for individual treatment stratification. O6-methylguanine-DNA methyltransferase (MGMT) is a DNA-repair protein that protects glioblastoma tumor cells against alkylating agents including TMZ by removing alkyl adducts from the O6-position of guanine.[1] The landmark European Organization for Research on Treatment Cancer (EORTC) 26981 trial [5]–[6], along with a series of confirmatory studies [7]–[10], had demonstrated that epigenetic silencing of MGMT gene by promoter methylation was of predictive significance for prolonged survival to the combination of TMZ and RT in younger glioblastoma patients (<70 years). Therefore, MGMT promoter methylation testing allowed the visualization of a promising future for highly individualized management of glioblastoma patients. However, its clinical implication is less defined in elderly population due to the frequent exclusion of this subpopulation from clinical studies.[11] The aim of our study is to systematically review literature data on the clinical relevance of MGMT promoter status in elderly glioblastoma patients, and to seek valid evidence for its determinant role for treatment stratification.

## Methods

### Trial eligibility

Studies investigating the association between *MGMT* promoter methylation status and survival data in elderly patients (≥ 65 years old) with newly diagnosed glioblastoma were eligible for inclusion. Different treatment modalities or schedules, different testing methods and different types of tumor samples were all included. Outcomes of interest included: 1) OS defined as the time interval from the date of diagnosis or randomization to the date of death or last follow-up; 2) progression-free survival (PFS), or the time interval from the date of diagnosis or randomization to the date of progression, which was defined by both clinical and radiologic criteria [12] or to the date of death or last follow-up without progression.

### Literature search

A systematic literature search was conducted using the PubMed, EMBASE and Cochrane Library, and no restrictions regarding publication date and language were applied. The following search strings were used 1) “glioma”, “glioblastoma”, “malignant glioma”, “high grade glioma”; 2) “elderly”, “older patients”, “advanced age”; 3) “O-6-methylguanine-DNA methyltransferase”, “MGMT”, “prognostic”, “biomarker”, “prediction”, “predictive”, “predictor”. Reference lists from related articles were also reviewed.

### Study selection and data extraction

Study selection was independently performed by two reviewers (YAA and ZLH) who were not blinded to study identity (e.g., authors, publication years, contact address) during eligibility assessment, and disputes were resolved through discussion. The newest publication of a same study was included. Data of interest were extracted using a piloted extraction sheet, as follows: authors, publication years, contact address, sample size, patients’ characteristics, types of specimens, assay methods, statistical methods and survival data.

### Assessment of the risk of bias in included studies

The risk of bias in each study was independently assessed by two reviewers (YAA and CJX) using a customized domain-based Newcastle Ottawa Scale (NOS). [13]–[14] The modified NOS covers the five major domains of possible bias of a given clinical study such as selection bias, performance bias, detection bias, attrition bias and reporting bias, and examines the important quality items identify by the Reporting Recommendations for Tumor Marker Prognostic Studies (REMARK) checklist for a tumor prognostic study. [15]–[16] The judgment criteria are specifically described in Table S1.

### Statistical analysis

Time-to-event data (e.g., OS, PFS) were analyzed using the hazard ratios (HRs) with a value less than one indicating favorable outcomes in elderly patients with methylated MGMT promoter or those with TMZ-containing therapies. If the HR was not directly reported, the value was calculated using the Kaplan-Meier survival curves or the methods reported by Tierney et al [17]. For proportions, data were computed using the logit transformation formula.[18] The inverse-variance method was used, and the application of either fixed- or random-effect model was based on between-study heterogeneity. The heterogeneity was estimated using Chi^2^ test and I^2^ statistic (the percentage of the variability in effect estimates that is due to heterogeneity rather than random error), with P*_heterogeneity_*<0.1 or I^2^>40% considered to be statistically significant.

Publication bias was assessed by visual impression, and was confirmed by analytic methods such as Egger’s test [19].

Both a subgroups analysis of studies with similar treatment and an interaction analysis between treatment and MGMT promoter status were implemented. A sensitivity analysis based on the assessment of risk of bias was also performed.

All analyses were done in R software v2.15.3 (R Foundation for Statistical Computing, Vienna, Austria) and Review Manager v5.2 (the Cochrane Collaboration, Software Update, Oxford, UK).

## Results

### Characteristics of included studies

**Figure 1:**
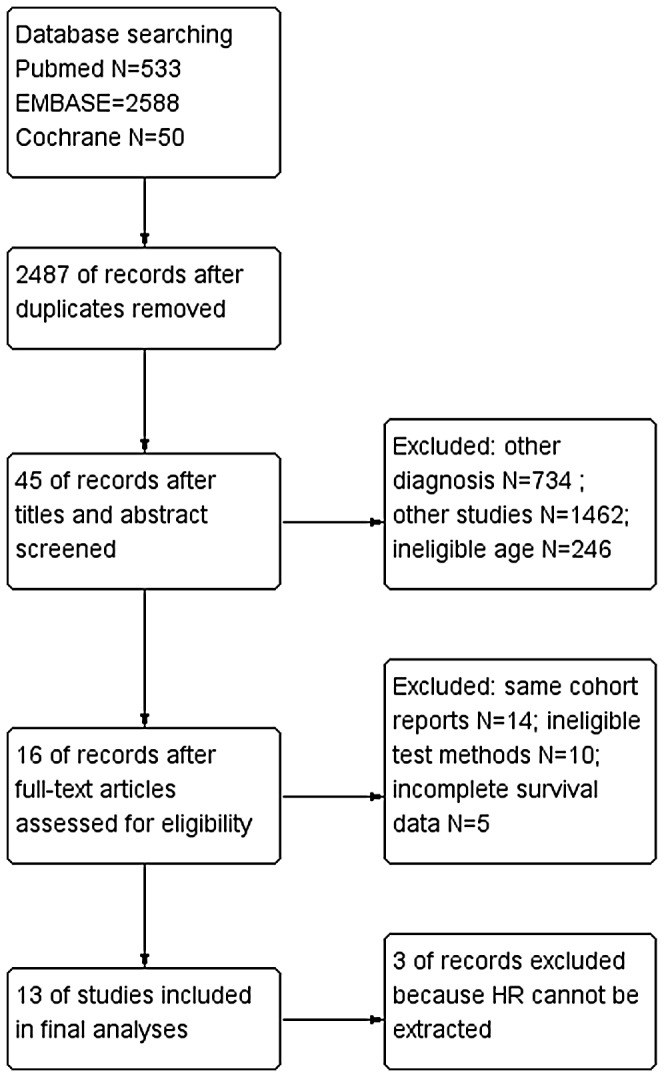
Flow diagram of study selection.

With the above eligibility criteria, 16 studies [20]–[35] were identified (2 phase III trials [24]–[25], 3 phase II trials [26], [31], [33], 4 prospective cohort studies [22]–[23], [34]–[35] and 7 retrospective cohort studies [20]–[21], [27]–[30], [32]; Fig. 1). Of those, 4 studies [20]–[22], [35] were published in abstract. Characteristics of the identified studies were summarized in Table 1.

**Table 1:** Characteristics of included studies.

First author (ref.)	Period of diagnosis	Cutoff age (yrs)	Testing methods	Treatment	Evaluable patients	MGMT status	Median OS	*P* value	Median PFS	*P* value
***Prospective studies***										
Philippe (22)	—	≥70	nr	RT+BCNU wafer	—	ME (—)	—	0.00	—	0.01
						UN (—)	—		—	
Reifenberger (23)	2004-2010	≥70	gel-based MSP	Supportive care	65	ME (34)	2.3 m	0.46	1.8 m	0.51
						UN (31)	2.0 m		1.7 m	
				RT	61	ME (31)	7.8 m	0.34	4.5 m	0.06
						UN (30)	8.8 m		5.2 m	
				TMZ	16	ME (14)	7.2 m	—	6.8 m	—
						UN (2)	2.6 m		0.5 m	
				RT+TMZ	91	ME (55)	13.1 m	0.01	7.3 m	0.14
						UN (36)	10.4 m		7.2 m	
Gellago Pérez-Larraya (26)	2007-2009	≥70	real-time MSP	TMZ	31	ME (13)	31wks	0.03	26.2wks	0.03
						UN (18)	18.7wks		11.1wks	
Malmström (24) *	2000-2009	≥60	real-time MSP	TMZ	72	ME (28)	9.7 m	0.02	—	—
						UN (44)	6.8 m		—	
				RT	131	ME (63)	8.2 m	0.81	—	—
						UN (68)	7.0 m		—	
Wick (25) *	2005-2009	≥65	real-time MSP #	TMZ	108	ME (31)	not reached	0.00	8.4 m	0.00
						UN (77)	7 m		3.3 m	
				RT	101	ME (42)	9.6 m	0.86	4.6 m	0.49
Brandes (31)	2004-2007	≥65	gel-based MSP	RT+TMZ	37	ME (16)	not reached	0.01	22.9 m	0.01
						UN (21)	13.7		9.5 m	
Minniti (33)	2005-2010	≥70	gel-based MSP	RT+TMZ	71	ME (32)	15.8 m	0.00	10 m	0.00
						UN (39)	8.8 m		4 m	
Fiorentino (34)	2001-2011	≥65	gel-based MSP	RT+TMZ	61	ME (24)	—	0.04	—	—
						UN (37)	—		—	
Franceschi (35)	2009-2010	≥70	nr	RT	26	ME (11)	8.8 m	0.55	—	—
						UN (15)	8 m		—	
				RT+TMZ	16	ME (6)	17.2 m	0.09	—	—
						UN (10)	8.5 m		—	
***Retrospective studies***										
Lombardi (20)	—	≥65	nr	RT+TMZ	43	ME (20)	—	—	—	—
						UN (23)	—		—	
Mishima (21)	2006	≥65	nr	RT+TMZ	23	ME (—)	25.8 m	0.05	—	—
						UN (—)	9.0 m		—	
Abhinav (27)	2007-2009	≥65	gel-based MSP	RT+TMZ	19	ME (9)	242d	0.25	—	—
						UN (10)	390d		—	
Gerstner (28)	1998-2009	≥70	gel-based MSP	RT+TMZ &	40	ME (24)	489d	0.00	405d	0.27
						UN (16)	263d		246d	
Piccirilli (29)	2000-2003	≥80	gel-based MSP	RT	10	ME (7)	16 m	0.10	10 m	0.09
						UN (3)	7 m		5 m	
				RT+TMZ	6	ME (5)	27 m	0.31	12 m	1.00
						UN (1)	15 m		12 m	
Sijben (30)	2004-2007	≥65	gel-based MSP	RT	13	ME (8)	—	0.73	—	0.86
						UN (5)	—		—	
				RT+TMZ	16	ME (5)	7.4 m	0.82	6.3 m	0.98
						UN (11)	8.5 m		6.0 m	
Minniti (32)	2005-2009	≥70	gel-based MSP	RT+TMZ	83	ME (42)	15.3 m	0.00	10.5 m	0.00
						UN (41)	10.2 m		5.5 m	

MSP = methylation-specific polymerase chain reaction; BCNU = carmustine; RT = radiotherapy; TMZ = temozolomide; MGMT = O6-methylguanine-DNA methyltransferase ME = methylatedtumors; UN = unmethylated tumors; OS = overall survival; PFS = progression-free survival; ref. = reference; nr = not reported; yr = year; m = month; wk = week; d = day.

randomized controlled trials.

^#^ gel-based MSP was also employed but not used to define MGMT promoter status.

^&^ also included chemotherapy alone (3 patients).

The cutoff age for elderly patients was defined variably across the studies, among which seven studies set the age cutoff at 65 years [20]–[21], [25], [27], [30]–[31], [34], seven studies set the cutoff at 70 years [22]–[23], [26], [28], [32]–[33], [35] and one study set the cutoff at 80 years [29]. Besides, one study with an inclusion criterion of 60 years or over was also included because most of their patients were eligible for our study [24]. Regarding the treatment, a number of adjuvant therapies were utilized in the studies such as supportive care, TMZ alone, RT alone and RT in combination with TMZ or carmustine wafer.[20]–[35]

Three studies were [20]–[22] finally excluded from the quantitative analyses, from which the HRs could not be extracted. Thus an assessment of risk of bias was performed on the remaining 13 studies using the customized domain-based NOS [23]–[35]. The assessment showed no apparent variations across the studies in most domains of bias, except for selection bias. Therefore, the risk of bias of the studies was ranked based on those variations: two randomized trials [24]–[25] were considered to be of lowest risk of bias, six [23], [26], [28]–[29], [31], [33] were of lower risk, and five [27], [30], [32], [34]–[35] were of higher risk (Table S2).

To explain explicitly the clinical impact of MGMT promoter status, the definition of the terms “prognostic” and “predictive” was used as follows: 1) a prognostic factor is a clinical or biologic characteristic that provides information on the likely outcome of the cancer disease independent of treatment; and 2) a predictive factor is a clinical or biologic characteristic that provides information on the likely benefit from one specific treatment rather than another (either in terms of tumor shrinkage or survival).[36]

### The presence of tumors with a methylated MGMT promoter

**Figure 2:**
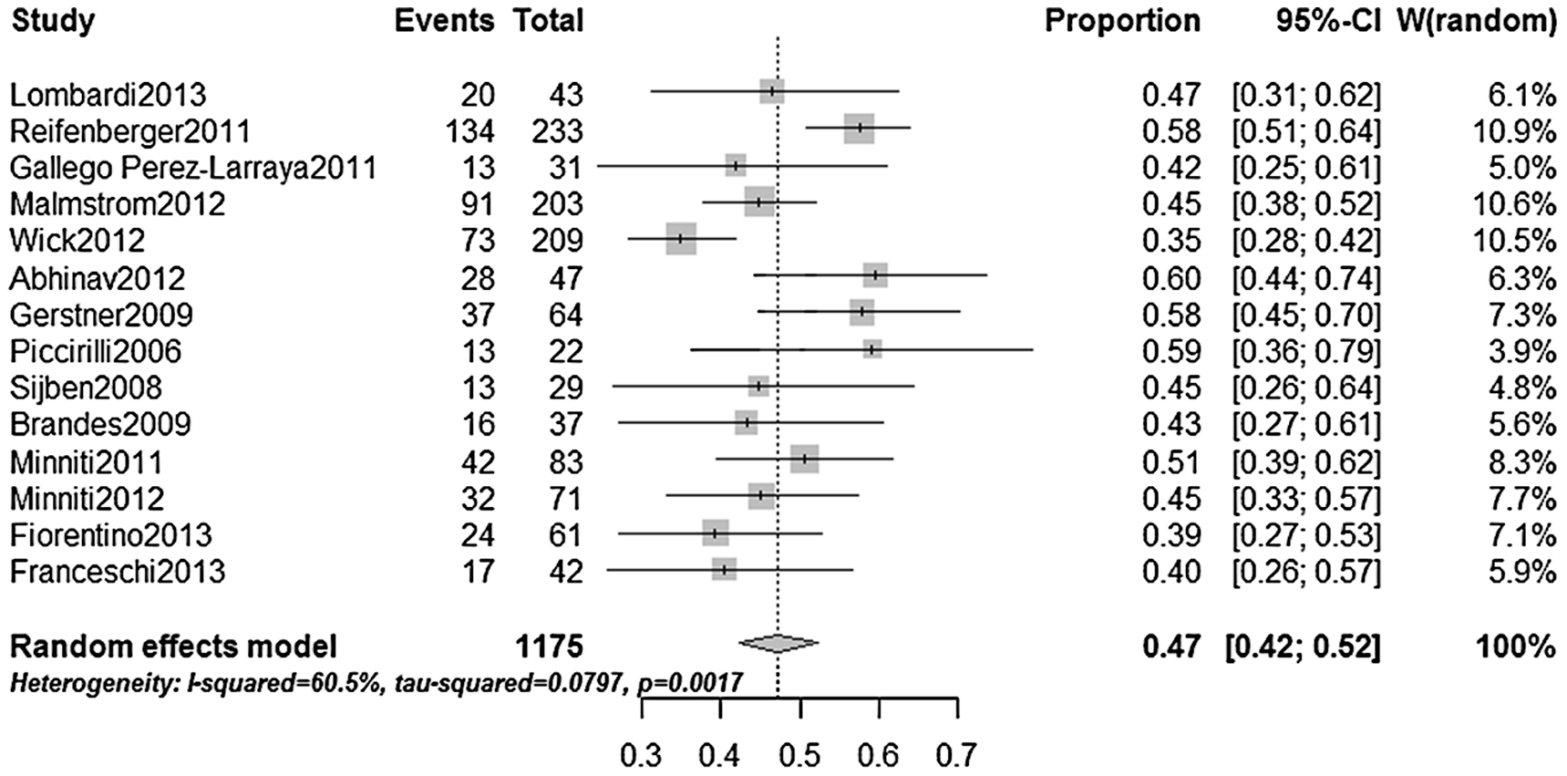
The aggregate estimate for the frequency of MGMT promoter methylation in elderly glioblastoma patients.

Promoter methylation status of MGMT was assessed using gel-based methylation-specific PCR (MSP) assays in 9 studies [23]–[34] and real-time MSP assays in 3 studies [24]–[26] (Table 1). The DNA samples were extracted from formalin-fixed, paraffin-embedded tumor tissues in most of the studies [24]–[34], except for one study (frozen tumor sections) [23]. Promoter methylation was defined according to the criteria of each study. Four studies in abstract did not report the methods of sample testing and handling. [20]–[22], [35] The documented presence of methylated MGMT promoter were 35%–60%, and an aggregate proportion was calculated as 47% with a 95% CI of 42–52%, using a randomeffect model (Fig. 2). The percentage were similar to the value for younger patients (the aggregate proportion: 44% with a 95% CI of 39–50%; data from individual reports ranged 19%–68% [37]), which indicated no apparent variations in the presence of methylated MGMT promoter by age.

### The prognostic value of MGMT promoter methylation status

**Figure 3:**
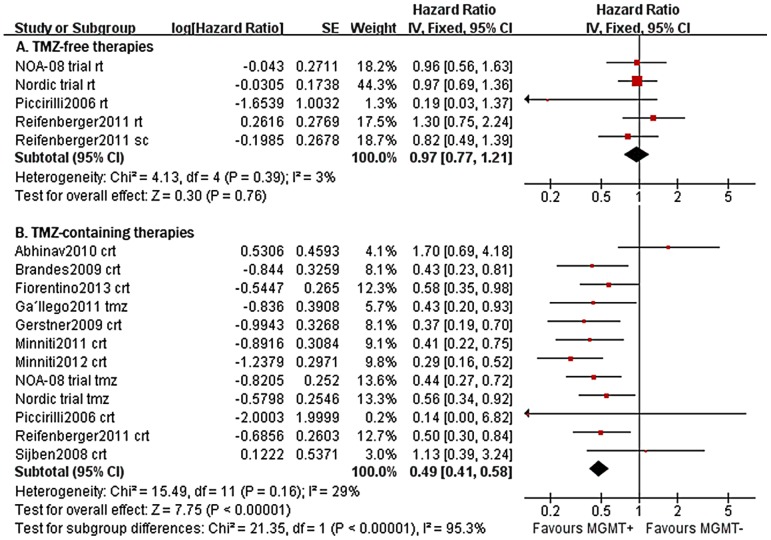
Forest plot of comparison. outcome: OS; comparison: methylated versus unmethylated: A. TMZ-free therapies; B. TMZ-containing therapies. (tmz = temozolomide; rt = radiotherapy; sc = supportive care; crt = chemoradiotherapy).

A random-effect meta-analysis of the studies with all treatments suggested a significant prognostic impact of MGMT promoter methylation status in older glioblastoma patients (methylated vs. unmethylated: 13 studies, 1119 patients; HR = 0.55, 95% CI 0.42–0.73; test for heterogeneity: Chi^2^ = 41.75, *P*<0.0001; I^2^ = 71%; Fig. S1) [23]–[34]. However, the subgroup analysis revealed apparent variations in HR estimations by each treatment (*P_subgroup analysis_* <0.00001; Fig. 3). The result showed that MGMT promoter methylation was associated with longer OS in elderly patients with TMZ-containing therapies (12 studies, 635 patients: HR = 0.49, 95% CI 0.41–0.58; test for heterogeneity: Chi^2^ = 15.49, *P* = 0.16, I^2^ = 29%; Fig. 3) but OS of patients with each promoter status did not show statistically significant difference when TMZ was withdraw (4 studies, 368 patients; HR = 0.97, 95% CI 0.77–1.21; test for heterogeneity: Chi^2^ = 4.13, *P* = 0.39, I^2^ = 3%; Fig. 3). The PFS analyses yielded a similar result with smaller simple size (9 studies, 747 patients, HR = 0.51, 95% CI 0.40–0.64; test for heterogeneity: Chi^2^ = 14.94, *P* = 0.06; I^2^ = 46%; Fig. S2). Overall, the presented information indicated that MGMT promoter methylation might not have a significant impact on survival of patients who were not treated by TMZ chemotherapy, and that the biomarker is less likely to have a universal prognostic significance in older glioblastoma patients regardless of the assigned treatments.

### The predictive value of MGMT promoter methylation status

**Figure 4:**
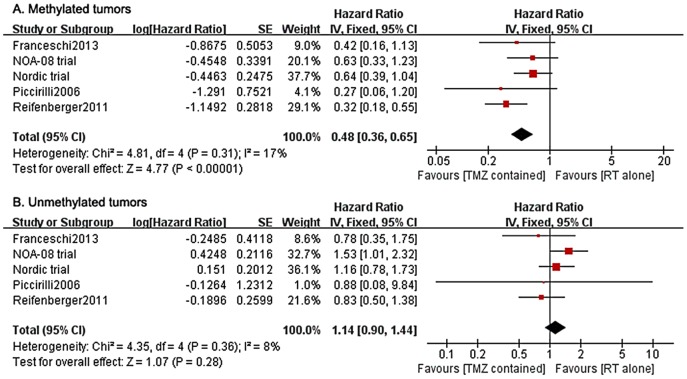
Forest plot of comparison. outcome: OS; comparison: TMZ-containing therapies versus TMZ-free therapies: A. methylated tumors; B. unmethylated tumors.

To investigate the predictive impact, we did an interaction analysis between the assigned treatments and MGMT promoter status. The analyses showed that, among patients with methylated tumors, TMZ-containing therapies were associated with longer OS compared with RT alone (5 studies, 293 patients: HR = 0.48, 95% CI 0.36–0.65; test for heterogeneity: Chi^2^ = 4.81, P = 0.31, I^2^ = 17%; Fig. 4A). By contrast, among those with unmethylated tumors, OS was not significantly improved with the addition of TMZ (5 studies, 345 patients: HR = 1.14, 95% CI 0.90–1.44; test for heterogeneity: Chi^2^ = 4.35, P = 0.36, I^2^ = 8%; Fig. 4B). Moreover, a subset analysis showed that TMZ alone was even inferior to RT alone in improving OS of patients with an unmethylated MGMT promoter (2 studies, 248 patients; HR = 1.32; 95% CI 1.00–1.76; test for heterogeneity: Chi^2^ = 0.88, P = 0.35, I^2^ = 0%; Table 2). The PFS analyses also supported the useful predictive value of this biomarker (Table 2). In summary, those results highlighted that MGMT promoter status could be a useful predictor for the response to TMZ-containing therapies and can help select older patients for optimal individualized treatment.

**Table 2:** Additional results of the subgroup and interaction analyses.

Treatment	Number of studies	Number of patients	Hazard ratio (95% CI)	P *_z-test_*	I^2^ statistic
***Subgroup analysis (methylated vs. unmethylated)***					
*Overall survival*					
1. TMZ-free therapies	4	368	0.97 [0.77, 1.21]	0.76	3%
Supportive care	1	65	0.82 [0.49, 1.39]	0.46	NA
RT	4	303	1.00 [0.78, 1.29]	0.98	18%
**2. TMZ-containing therapies**	**12**	**635**	**0.49 [0.41, 0.58]**	**0.00**	**29%**
**TMZ**	**3**	**211**	**0.48 [0.35, 0.67]**	**0.00**	**0%**
**RT/TMZ**	**9**	**424**	**0.51 [0.37, 0.70]**	**0.00**	**46%**
*Progression-free survival*					
1. TMZ-free therapies	3	237	0.97 [0.59, 1.57]	0.89	58%
Supportive care	1	65	0.84 [0.50, 1.42]	0.51	NA
RT	3	172	0.97 [0.45, 2.07]	0.94	70%
**2. TMZ-containing therapies**	**9**	**477**	**0.49 [0.40, 0.60]**	**0.00**	**15%**
**TMZ**	**2**	**139**	**0.36 [0.25, 0.52]**	**0.00**	**0%**
**RT/TMZ**	**7**	**338**	**0.53 [0.44, 0.65]**	**0.00**	**0%**
***Interaction analysis between treatment and MGMT status***					
*Overall survival*					
1. Methylated tumors					
**TMZ-containing vs. RT**	**5**	**293**	**0.48 [0.36, 0.65]**	**0.00**	**17%**
**TMZ vs. RT**	**3**	**209**	**0.66 [0.47, 0.93]**	**0.02**	**0%**
**RT/TMZ vs. RT**	**3**	**115**	**0.33 [0.21, 0.52]**	**0.00**	**0%**
2. Unmethylated tumors					
TMZ-containing vs. RT	5	345	1.14 [0.90, 1.44]	0.28	8%
**TMZ vs. RT**	**2**	**248**	**1.32 [1.00, 1.76]**	**0.05**	**0%**
RT/TMZ vs. RT	3	95	0.80 [0.51, 1.27]	0.35	0%
*Progression-free survival*					
1. Methylated tumors					
**TMZ-containing vs. RT**	**3**	**187**	**0.35 [0.20, 0.62]**	**0.00**	**45%**
**TMZ vs. RT**	**2**	**118**	**0.49 [0.32, 0.74]**	**0.00**	**0%**
**RT/TMZ vs. RT**	**2**	**98**	**0.23 [0.07, 0.70]**	**0.01**	**46%**
2. Unmethylated tumors					
TMZ-containing vs. RT	3	208	1.08 [0.42, 2.78]	0.87	82%
**TMZ vs. RT**	**1**	**136**	**2.11 [1.47, 3.02]**	**0.00**	**NA**
RT/TMZ vs. RT	2	70	0.71 [0.41, 1.23]	0.22	30%

RT = radiotherapy; TMZ = temozolomide; MGMT = O6-methylguanine-DNA methyltransferase; CI = confidence interval; NA = not applicable; TMZ-free therapies = RT alone and supportive care; TMZ-contained therapies = TMZ alone and combined RT/TMZ.

In bold type were reported statistically significant results.

The results from the subgroup and interaction analyses were presented in Table 2.

### Assessment of publication bias

Visual impression of the funnel plots of both the subgroup and interaction analyses indicated absence of publication bias, which was confirmed by Egger’s test. The results were presented in Table S3.

### Sensitivity analysis

Based on the risk assessment by the modified NOS, a sensitivity analysis was conducted only analyzing the studies with lower or lowest risk in the domain of selection bias, and yielded consistent results with the primary findings. The results were summarized in Table S4.

## Discussion

The optimal management for elderly glioblastoma patients remains elusive due to the absence of validated data from clinical studies and the great heterogeneity of this fragile subpopulation in terms of physical condition, co-morbidity status, treatment tolerance and clinical prognosis.[2], [11] Thus clinically relevant biomarkers are needed to individualize the treatment. The DNA-repair enzyme MGMT conferred the major resistance to alkylating agents in glioblastoma patients[38], and epigenetic silencing of MGMT by gene promoter methylation had been widely investigated in younger glioblastoma patients.[38] However, the clinical relevance is ill-defined in older patients.[6] The present study, first of all, reports a stable presence of methylated MGMT promoter in older patient cohort, indicating a less possibility of an altered MGMT methylation pattern that contributes to the poorer prognosis of elderly glioblastoma patients. Second, the study suggests that MGMT promoter status might not have a treatment-independent prognostic value in older glioblastoma patients, because it did not have a significant impact on survival of those without TMZ chemotherapy. Therefore, methylated MGMT promoter alone is less likely to be associated with underlying genetic or epigenetic alterations that molecularly define a more favorable glioblastoma subtype. Finally and most importantly, the present study highlights a useful predictive role of MGMT promoter status for a better response to TMZ chemotherapy. The results showed that among patients with methylated tumors, TMZ-containing therapies conferred a clear survival benefit as compared to RT alone, whereas among those with unmthylated tumors, they seemed not to be more beneficial than RT alone. Moreover, TMZ alone seemed to be even inferior to RT alone in improving survival. In summary, the meta-analysis highlights the therapeutic implications of MGMT promoter status for a better treatment choice of elderly glioblastoma patients.

Assessment of risk of bias in included studies using quality evaluation tools is a reliable way to control the possible bias from study design, performance and reporting in a meta-analysis.[15] However, fewer evaluation tools had been justified for assessment of a tumor prognostic study, which in nature is a non-randomized study because participants are not possible to be randomized to the groups with different biomarker statues.[16] Recently, a novel domain-based NOS was proposed as a potential helpful and practical method.[14] The novel NOS was modified through (1) completing all the domains of possible bias in a given clinical study; (2) assigning specific responses, such as “yes”, “no” or “unclear”, instead of stars, to each item; and (3) ranking the risk of bias of each study according to its overall responses, rather than the scoring of stars (Table S1). In this study, the tool was further customized to fit the topic of this review through incorporating the important quality items from the REMARK guideline, which was originally developed to standardize the reporting of a tumor prognostic study.[16] Of note, all modifications were done according to the recommendations from the Cochrane Non-Randomised Studies Methods Group (NRSMG).[15] Based on the results of the risk assessment, a sensitivity analysis was conducted only analyzing the studies with lower or lowest risk in the domain of selection bias, and showed consistent results (Table S4). Therefore, our findings can be regarded with a higher degree of certainty. However, it must be acknowledged that the novel NOS has not been fully validated and we should interpret the results with caution.

Promoter status of the MGMT has been established as a strong clinically relevant factor in glioblastoma patients, the mandatory testing of this biomarker in routine practice is however highly controversial, because insufficient data were by far available to justify a direct conclusion between MGMT status testing and individual treatment choice, especially for younger patients.[39] In a *post hoc* analysis of the EORTC 26981 trial [6], the combination of RT and TMZ conferred a modest but significant survival benefit in younger patients (<70 years) who had an unmethylated MGMT promoter. Therefore, given the absence of effective alternative therapies and the good tolerance to the aggressive combined treatment, adjuvant TMZ chemotherapy was not likely to be withheld from the standard care for this subset of younger patients. The therapeutic implication of this biomarker testing was much compromised in younger patients.[39] By contrast, MGMT testing could be more informative for elderly patients who were featured with poor physical conditions, complicated comorbid disorders and decreased treatment tolerance. In our review, TMZ-containing therapies failed to show additional survival benefits as compared to RT alone in elderly patients with an unmethylated MGMT promoter (Table 2). It was known that, among the published literature, TMZ chemotherapy was associated with a notable number of grade 3–4 toxicities in elderly patients, in whom, even mild toxicities can decrease quality of life and treatment compliance.[24]–[25], [30], [32]–[33] Therefore, TMZ is very likely to be withdrawn from the standard of care for those with an unmethylated MGMT promoter, and alternatively RT alone can be a more reasonable option for them. In summary, the presented data indicated a direct association between MGMT testing and individual treatment decision especially in older patients. Importantly, data on MGMT are being collected in a number of randomized trials which evaluates the combination of novel targeted agents (e.g., the integrin antagonist cilengitide, the epidermal growth factor receptor antibody nimotuzumab) to standard treatment in younger patients.[40] If the predictive significance of MGMT status can be confirmed in those trials, the routine MGMT testing will be recommended for all patients.

The provocative clinical relevance of MGMT promoter status has led to an ongoing debate over the establishment of a standardized test method, which is suitable for high-throughput analysis from small amounts of DNA samples (e.g., formalin-fixed, paraffin-embedded tumor tissues) and highly reproducible in independent laboratories.[37] Two assay methods, i.e., gel-based and real-time MSP, were used in the included studies. The conventional, qualitative gel-based MSP had established the predictive value of MGMT promoter status in glioblastoma patients, and was widely used in clinical trials.[37] This methodology however has major drawbacks for routine clinical utility, such as inability to detect irregular mosaic methylation patterns, susceptibility to environmental contamination, considerable intra-laboratory variability and post-PCR time and labor.[39], [41]–[42] Moreover, the gel-based readout cannot provide clear cutoff for the determination of a methylated promoter.[37] The direct, real-time MSP assay is the current preferred method which yields a quantitative test result by normalizing the copy number of a methylated MGMT promoter to a control gene.[43] Compared with the conventional gel-based assay, the novel test protocol is considered of having higher sensitivity, higher reproducibility and higher efficiency due to the real-time PCR platform [42]–[43]. A quantitative readout can also allow the determination and investigation of an optimal cutoff point for clinical prediction.[37] The new assay is now being used to stratify patients in most clinical trials for glioblastoma (e.g., the cilengitide trial mentioned, RTOG 0825 trial, RTOG 0525 trial). However, despite substantial improvement, the novel test still has technical limitations (e.g., incomplete bisulfite conversion, nontumoral tissue contamination, a prospectively unvalidated cut-off point).[37], [41], [43] Besides, variations in pre-analytic tumor tissue handling can invariably bring further uncertainty into the interpretation of their results.[39] Newest technology (e.g., prosequencing, methylation-specific multiplex ligation-dependent probe) is being carefully evaluated to overcome the above drawbacks.[37] Disappointedly, no consensus has been reached on a generally accepted method by far due to the lack of studies specifically comparing the merits and disadvantages of different testing protocols.[39] Therefore, before the wide application of the MGMT promoter test, we will await to identify a standardized, efficient and reproducible testing protocol.

The presented information should be interpreted carefully because some limitations existed. First, data on MGMT promoter status were only available for a selected patient subgroup of overall trial population which can induce selection bias in the analysis. Second, with limited data on PFS, the influence of salvage treatment at progression cannot be ruled out. Third, the predictive significance of MGMT status was not always validated in a controlled, prospective manner (e.g., the predictive role in the group’s comparison of RT/TMZ vs. RT alone), which is the only way to demonstrate the predictive value of a biomarker, and its prognostic effect was not always being studied in a group of patients free from systematic adjuvant treatment. Finally, even in a non-optimal trial design, the distributions of patient prognostic variables were not always being adjusted (e.g., by using multivariate modeling or matched control groups) and MGMT endpoints were usually secondary outcome with statistical under-powering issues (Table S2).

## Conclusion

### Implication for practice

The meta-analysis encouraged the mandatory testing of MGMT promoter status in routine practice in elderly glioblastoma patients due to the observation of a strong predictive but not prognostic value of this biomarker to TMZ chemotherapy and a direct association between MGMT testing and individual treatment choice.

### Implication for research

Future studies of MGMT molecular analysis are needed 1) to validate its predictive value for the comparison of combined RT/TMZ vs. RT alone in older patients and 2) to justify its therapeutic implications for younger patients. Furthermore to optimize the personalized treatment of elderly ones, other clinically relevant factors (e.g., KPS, co-morbidities or other molecular biomarkers) are needed to further stratify the elderly with methylated tumors for treatment choice between combined RT/TMZ and TMZ alone. Finally, the modification and standardization of MGMT testing approaches are much needed in future.

## Supporting Information

Figure S1 **Forest plot of comparison: outcome: OS; comparsion: methylated versus unmethylated: all treatments included.**
(TIF)

Figure S2 **Forest plot of comparison: outcome: PFS; comparsion: methylated versus unmethylated: all treatments included.**
(TIF)

Checklist S1 **PRISMA Checklist.**
(DOC)

Table S1 **Criteria for judgment of risk of bias in the modified domain-based Newcastle-Ottawa Scale (NOS).**
(DOC)

Table S2 **Assessment of risk of bias of included studies.**
(DOC)

Table S3 **Egger’s test for publication bias.**
(DOC)

Table S4 **The results of the sensitivity analysis.**
(DOC)
