# Supplementary figures and images for: The Predictive but Not Prognostic Value of MGMT Promoter Methylation Status in Elderly Glioblastoma Patients: A Meta-Analysis
Source: PLoS One. 2014 Jan 13;9(1):e85102. doi: 10.1371/journal.pone.0085102 (PMC3890309; doi:10.1371/journal.pone.0085102)

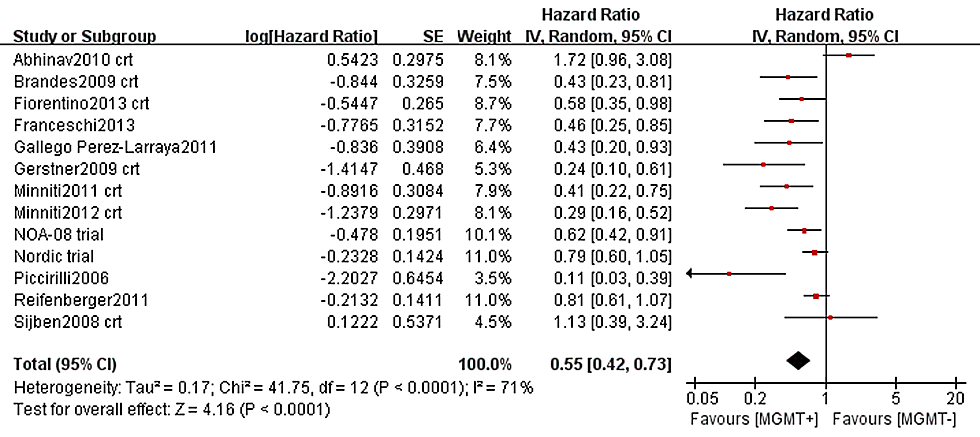

Supplement: Figure S1 — Forest plot of comparison: outcome: OS; comparsion: methylated versus unmethylated: all treatments included. (TIF) [file pone.0085102.s001.tif]

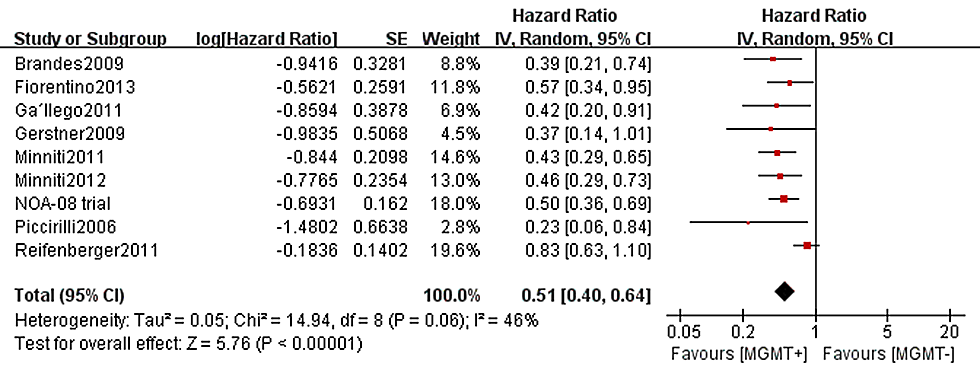

Supplement: Figure S2 — Forest plot of comparison: outcome: PFS; comparsion: methylated versus unmethylated: all treatments included. (TIF) [file pone.0085102.s002.tif]
