# Supplementary material for: The Predictive but Not Prognostic Value of MGMT Promoter Methylation Status in Elderly Glioblastoma Patients: A Meta-Analysis
Source: PLoS One. 2014 Jan 13;9(1):e85102. doi: 10.1371/journal.pone.0085102 (PMC3890309; doi:10.1371/journal.pone.0085102)
Supplement: Table S1 — Criteria for judgment of risk of bias in the modified domain-based Newcastle-Ottawa Scale (NOS). (DOC) [file pone.0085102.s004.doc]

**Supplemental Material Table S1: Criteria for judgment of risk of bias in the modified domain-based Newcastle-Ottawa Scale (NOS) [1-3]**

| ***Domains*** | **Judgment criteria for responses to each domain** | | |  |
| --- | --- | --- | --- | --- |
|  | ***Yes*** | ***No*** | ***Unclear*** |  |
| ***Selection bias*** |  |  |  |  |
| *Selection* |  |  |  |  |
| Treatment assignment: randomization? | *The investigators describe a random component in the sequence generation process such as coin tossing, using a computer random number generator, etc.* | *The investigators describe a non-random component in the sequence generation process such as sequence generated based on hospital or clinical record number, allocation by judgment of the clinician, etc.* | *Insufficient description* |  |
| How representative was the patient selection in comparison with the general elderly patients with GBMs? | *Truly or somewhat representative of the general elderly population with GBMs* | *Selected group of e.g. doctors, nurses* | *Insufficient description* |  |
| *Comparability* |  |  |  |  |
| State the distribution of the following important prognostic variables (age, gender, KPS or ECOG performance score, surgery, adjuvant treatment, co-morbidity, tumor location or numbers; neurological status) between the groups with different MGMT statues | *Most of the variables (e.g., at least 4 out of 8 items) were reported* | *At least two variables were not reported* | *Not applicable* |  |
| Groups comparable for the abovementioned variables | *All reported variables were comparable between the groups* | *At least one of those was not comparable* | *Comparability was not applicable because insufficient data were reported* |  |
| Methods applied for controlling the potential prognostic confounders | *Appropriate methods are used to control the potential confounders (e.g. multivariate modeling, matching, etc)* | *No method was applied to control the potential confounders* | *Insufficient description* |  |
| ***Performance bias*** |  |  |  |  |
| *Performance* |  |  |  |  |
| State statistical sample size or power calculation | *Reported* | *Not reported* | *Not applicable* |  |
| Blinding of participants and personnel | *1. No blinding or incomplete blinding, but the reviewers judge that the outcome is not likely to be influenced by lack of blinding*  *2. Blinding of key study participants and personnel and unlikely that the blinding could been broken* | *1. No blinding or incomplete blinding, the outcome is likely to be influenced by lack of blinding*  *2. Blinding of key study participants and personnel and likely that the blinding could been broken, and the outcome is likely to be influenced by lack of blinding* | *Insufficient description or the study did not address the outcome* |  |
| Ascertainment of intervention exposure | *Medical records or structured interview* | *Written self report* | *Insufficient description* |  |
| *Assay method* |  |  |  |  |
| The method of sample handling | *Reported* | *Not reported* | *Not applicable* |  |
| The type of assay methods used | *Reported* | *Not reported* | *Not applicable* |  |
| Cutoff point determination | *Reported* | *Not reported* | *Not applicable* |  |
| ***Detection bias*** |  |  |  |  |
| Blinding of outcome assessment | *1. No blinding of outcome assessment, but the reviewers judge that the outcome measurement is not likely to be influenced by lack of blinding.*  *2. Blinding of outcome assessment ensured, and unlikely that the blinding could have been broken.* | *1. No blinding of outcome assessment, the outcome measurement is likely to be influenced by lack of blinding.*  *2. Blinding of outcome assessment ensured, and likely that the blinding could have been broken and the outcome assessment is likely to be influenced by lack of blinding.* | *Insufficient description or the study did not address the outcome* |  |
| Ascertainment of outcome data | *Record linkage* | *Self report* | *Insufficient description* |  |
| ***Attrition bias*** |  |  |  |  |
| Adequacy of outcome data | *1. The follow-up was long enough for outcomes to occur*  *2. Adequate follow up (e.g.≥80%) or subjects lost to follow up unlikely to introduce bias (e.g. for survival data, censoring unlikely to be introducing bias )* | *1. The follow-up was not long enough for outcomes to occur*  *2. Inadequate follow up (e.g.<80%)or subjects lost to follow up are very likely to introduce bias (e.g. for dichotomous outcome data, the proportion of missing outcomes compared with observed event risk enough to induce bias)* | *Insufficient description or the study did not address the outcome* |  |
| ***Reporting bias*** |  |  |  |  |
| Selective outcome reporting | *1. the study protocol is available and all of the study’s pre-specified outcomes of interest in the review have been reported in the pre-specified way*  *2. the study protocol is not available but it is clear that the published reports included all expected outcomes* | *1. not all of pre-specified outcomes have been reported*  *2. one or more outcomes were reported in a way that were not pre-specified*  *3. one or more outcomes were reported incompletely so that they cannot be entered in a meta-analysis*  *4. the study report failed to include results for a key outcome that would be expected to have been reported for such a study* | *Insufficient description** |  |

RT=radiotherapy; TMZ=temozolomide; GBM: glioblastoma; KPS= Karnofsky performance status; MGMT=O6-methylguanine-DNA methyltransferase

*It is very likely that the majority of studies will fall into this category, especially for those that the study protocol is not available.

**References:**

1. Higgins JP, Green S (2012) Cochrane Handbook for Systematic Reviews of Interventions: http://handbook.cochrane.org/.

2. Wells G, Shea B, O'Connell D, Peterson J, Welch V, et al. (2012) The Newcastle-Ottawa Scale (NOS) for assessing the quality of nonrandomised studies in meta-analyses.: http://www.ohri.ca/programs/clinical_epidemiology/oxford.asp.

3. Altman DG, McShane LM, Sauerbrei W, Taube SE (2012) Reporting Recommendations for Tumor Marker Prognostic Studies (REMARK): explanation and elaboration. PLoS Med 9: e1001216.
