# Supplementary material for: The Predictive but Not Prognostic Value of MGMT Promoter Methylation Status in Elderly Glioblastoma Patients: A Meta-Analysis
Source: PLoS One. 2014 Jan 13;9(1):e85102. doi: 10.1371/journal.pone.0085102 (PMC3890309; doi:10.1371/journal.pone.0085102)
Supplement: Table S2 — Assessment of risk of bias of included studies. (DOC) [file pone.0085102.s005.doc]

**Supplemental Material Table S2: Assessment of risk of bias of included studies**

| ***Domains*** | Reifenberger | Gellago Pe´rez | Malmström | Wick | Abhinav | Gerstner | Piccirlli |
| --- | --- | --- | --- | --- | --- | --- | --- |
|  | 2012 [23] | -Larraya 2011 [26] | 2012 [24] | 2012 [25] | 2013 [27] | 2009 [28] | 2006 [29] |
| ***Selection bias*** |  |  |  |  |  |  |  |
| *1. Selection* |  |  |  |  |  |  |  |
| 1.1 Treatment assignment: randomization? | *No* | *No* | *Yes* | *Yes* | *No* | *No* | *No* |
| 1.2 Method of patient selection or data collection: prospective? | *Yes* | *Yes* | *Yes* | *Yess2-to-event datastrospective?0000000000000000000000000000000000000000000000000000000000000000000000000000000000000000000000000* | *No* | *No* | *No* |
| 1.3 How representative was the patient selection in comparison with the general elderly patients with GBMs? | *Yes* | *Yes* | *Yes* | *Yes* | *Yes* | *Yes* | *Yes* |
| *2. Comparability* |  |  |  |  |  |  |  |
| 2.1 State the distribution of important prognostic variables between the groups? | *No* | *No* | *No* | *No* | *No* | *Yes* | *Yes* |
| 2.2 Group comparable for the reported variables? | *Unclear* | *Unclear* | *Unclear* | *Unclear* | *Unclear* | *Yes* | *Yes* |
| 2.3 Methods applied for controlling the potential prognostic confounders | *Yes* | *Yes* | *Yes* | *Yes* | *No* | *Yes* | *No* |
| ***Performance bias*** |  |  |  |  |  |  |  |
| 3. Performance |  |  |  |  |  |  |  |
| 3.1 State statistical sample size or power calculation | *No* | *No* | *No* | *No* | *No* | *No* | *No* |
| 3.2 Blinding of participants and personnel (time-to-event data)* | *Yes* | *Yes* | *Yes* | *Yes* | *Yes* | *Yes* | *Yes* |
| 3.3 Exposure of interventions | *Yes* | *Yes* | *Yes* | *Yes* | *Yes* | *Yes* | *Yes* |
| *4. Assay method* |  |  |  |  |  |  |  |
| 4.1 The method of sample handling | *Yes* | *Yes* | *Yes* | *Yes* | *Yes* | *Yes* | *Yes* |
| 4.2 The type of assay methods used | *Yes* | *Yes* | *Yes* | *Yes* | *Yes* | *Yes* | *Yes* |
| 4.3 Cutoff point determination^#^ | *Yes* | *Yes* | *Yes* | *Yes* | *Yes* | *Yes* | *Yes* |
| ***Detection bias*** |  |  |  |  |  |  |  |
| 5. Blinding of outcome assessment (time-to-event data)* | *Yes* | *Yes* | *Yes* | *Yes* | *Yes* | *Yes* | *Yes* |
| 6. Ascertainment of outcome data | *Yes* | *Yes* | *Yes* | *Yes* | *Yes* | *Yes* | *Yes* |
| ***Attrition bias*** |  |  |  |  |  |  |  |
| 7.1 Adequacy of outcome data (OS) | *Yes* | *Yes* | *Yes* | *Yes* | *Yes* | *Yes* | *Yes* |
| 7.2 Adequacy of outcome data (PFS) | *Yes* | *Yes* | *Unclear* | *Yes* | *Unclear* | *Yes* | *Yes* |
| ***Reporting bias*** |  |  |  |  |  |  |  |
| 8. Selective outcome reporting | *Unclear* | *Unclear* | *No* | *No* | *Unclear* | *Unclear* | *Unclear* |

| ***Domains*** | Sijben | Brandes | Minniti | Minniti | Fiorentino | Franceschi |
| --- | --- | --- | --- | --- | --- | --- |
|  | 2008 [30] | 2009 [31] | 2011 [32] | 2012 [33] | 2013 [34] | 2013 [35] |
| ***Selection bias*** |  |  |  |  |  |  |
| *1. Selection* |  |  |  |  |  |  |
| 1.1 Treatment assignment: randomization? | *No* | *No* | *No* | *No* | *No* | *No* |
| 1.2 Method of patient selection or data collection: prospective? | *No* | *Yes* | *No* | *Yes* | *Yes* | *Yes* |
| 1.3 How representative was the patient selection in comparison with the general elderly patients with GBMs? | *Yes* | *Yes* | *Yes* | *Yes* | *Yes* | *Yes* |
| *2. Comparability* |  |  |  |  |  |  |
| 2.1 State the distribution of important prognostic variables between the groups? | *No* | *No* | *No* | *No* | *No* | *No* |
| 2.2 Group comparable for the reported variables? | *Unclear* | *Unclear* | *Unclear* | *Unclear* | *Unclear* | *Unclear* |
| 2.3 Methods applied for controlling the potential prognostic confounders | *No* | *Yes* | *Yes* | *Yes* | *No* | *Unclear* |
| ***Performance bias*** |  |  |  |  |  |  |
| 3. Performance |  |  |  |  |  |  |
| 3.1 State statistical sample size or power calculation | *No* | *No* | *No* | *No* | *No* | *No* |
| 3.2 Blinding of participants and personnel (time-to-event data)* | *Yes* | *Yes* | *Yes* | *Yes* | *Yes* | *Yes* |
| 3.3 Exposure of interventions | *Yes* | *Yes* | *Yes* | *Yes* | *Yes* | *Yes* |
| *4. Assay method* |  |  |  |  |  |  |
| 4.1 The method of sample handling | *Yes* | *Yes* | *Yes* | *Yes* | *No* | *No* |
| 4.2 The type of assay methods used | *Yes* | *Yes* | *Yes* | *Yes* | *Yes* | *No* |
| 4.3 Cutoff point determination^#^ | *Yes* | *Yes* | *Yes* | *Yes* | *Yes* | *No* |
| ***Detection bias*** |  |  |  |  |  |  |
| 5. Blinding of outcome assessment (time-to-event data)* | *Yes* | *Yes* | *Yes* | *Yes* | *Yes* | *Yes* |
| 6. Ascertainment of outcome data | *Yes* | *Yes* | *Yes* | *Yes* | *Yes* | *Yes* |
| ***Attrition bias*** |  |  |  |  |  |  |
| 7.1 Adequacy of outcome data (OS) | *Yes* | *Yes* | *Yes* | *Yes* | *Yes* | *Yes* |
| 7.2 Adequacy of outcome data (PFS) | *Unclear* | *Yes* | *Yes* | *Yes* | *Yes* | *Unclear* |
| ***Reporting bias*** |  |  |  |  |  |  |
| 8. Selective outcome reporting | *Unclear* | *Unclear* | *Unclear* | *Unclear* | *Unclear* | *Unclear* |

* Yes was assigned despite that blinding was not conducted because the reviewers authors judged that OS and PFS were unlikely to be influenced by lack of blinding.

^#^ Yes was assigned if the assay methods applied had qualitatively binary readouts.
