# Supplementary material for: The Predictive but Not Prognostic Value of MGMT Promoter Methylation Status in Elderly Glioblastoma Patients: A Meta-Analysis
Source: PLoS One. 2014 Jan 13;9(1):e85102. doi: 10.1371/journal.pone.0085102 (PMC3890309; doi:10.1371/journal.pone.0085102)
Supplement: Table S3 — Egger’s test for publication bias. (DOC) [file pone.0085102.s006.doc]

**Supplementary Material Table S3: Egger’s test for publication bias**

| Overall survival | Included studies | P value for Egger's test |
| --- | --- | --- |
| **Subgroup analysis** |  |  |
| TMZ-free therapies | 4 | 0.23 |
| TMZ-containing therapies | 12 | 0.70 |
| **Interaction analysis** |  |  |
| TMZ containing vs. RT alone |  |  |
| Methylated tumors | 5 | 0.54 |
| Unmethylated tumors | 5 | 0.46 |

TMZ=temozolomide; RT=radiotherapy.
