# Supplementary material for: The Predictive but Not Prognostic Value of MGMT Promoter Methylation Status in Elderly Glioblastoma Patients: A Meta-Analysis
Source: PLoS One. 2014 Jan 13;9(1):e85102. doi: 10.1371/journal.pone.0085102 (PMC3890309; doi:10.1371/journal.pone.0085102)
Supplement: Table S4 — The results of the sensitivity analysis. (DOC) [file pone.0085102.s007.doc]

**Supplementary Material Table S4: The results of the sensitivity analysis***

| Treatment | Number of studies | Hazard ratio  (95% CI) | P *_z-test_* | I^2^ statistic |
| --- | --- | --- | --- | --- |
| ***Methylated vs. unmethylated*** | |  |  |  |
| *Overall survival* | |  |  |  |
| 1. TMZ-free therapies | 4 | 0.97 [0.77, 1.21] | 0.76 | 3% |
| Supportive care | 1 | 0.82 [0.49, 1.39] | 0.46 | NA |
| RT | 4 | 1.00 [0.78, 1.29] | 0.98 | 18% |
| **2. TMZ-containing therapies** | **8** | **0.43 [0.35, 0.54]** | **0.00** | **0%** |
| **TMZ** | **3** | **0.48 [0.35, 0.67]** | **0.00** | **0%** |
| **RT/TMZ** | **5** | **0.40 [0.30, 0.53]** | **0.00** | **0%** |
| *Progression-free survival* | |  |  |  |
| 1. TMZ-free therapies | 3 | 0.97 [0.59, 1.57] | 0.89 | 58% |
| Supportive care | 1 | 0.84 [0.50, 1.42] | 0.51 | NA |
| RT | 3 | 0.97 [0.45, 2.07] | 0.94 | 70% |
| **2. TMZ-containing therapies** | **6** | **0.48 [0.38, 0.59]** | **0.00** | **28%** |
| **TMZ** | **2** | **0.36 [0.25, 0.52]** | **0.00** | **0%** |
| **RT/TMZ** | **4** | **0.55 [0.42, 0.72]** | **0.00** | **9%** |
| ***Interaction analysis*** |  |  |  |  |
| *Overall survival* |  |  |  |  |
| 1. Methylated tumors |  |  |  |  |
| **TMZ-containing vs. RT** | **4** | **0.49 [0.36, 0.67]** | **0.00** | **37%** |
| **TMZ vs. RT** | **3** | **0.66 [0.47, 0.93]** | **0.02** | **0%** |
| **RT/TMZ vs. RT** | **2** | **0.31 [0.18, 0.52]** | **0.00** | **0%** |
| 2. Unmethylated tumors |  |  |  |  |
| TMZ-containing vs. RT | 4 | 1.18 [0.92, 1.51] | 0.19 | 13% |
| **TMZ vs. RT** | **2** | **1.32 [1.00, 1.76]** | **0.05** | **0%** |
| RT/TMZ vs. RT | 2 | 0.81 [0.46, 1.43] | 0.47 | 0% |
| *Progression-free survival* |  |  |  |  |
| 1. Methylated tumors |  |  |  |  |
| **TMZ-containing vs. RT** | **3** | **0.35 [0.20, 0.62]** | **0.00** | **45%** |
| **TMZ vs. RT** | **2** | **0.49 [0.32, 0.74]** | **0.00** | **0%** |
| **RT/TMZ vs. RT** | **2** | **0.23 [0.07, 0.70]** | **0.01** | **46%** |
| 2. Unmethylated tumors |  |  |  |  |
| TMZ-containing vs. RT | 3 | 1.08 [0.42, 2.78] | 0.87 | 82% |
| **TMZ vs. RT** | **1** | **2.11 [1.47, 3.02]** | **0.00** | **NA** |
| RT/TMZ vs. RT | 2 | 0.71 [0.41, 1.23] | 0.22 | 30% |

RT=radiotherapy; TMZ=temozolomide; MGMT=O6-methylguanine-DNA methyltransferase; CI=confidence interval; NA=not applicable; TMZ-free therapies=RT alone and supportive care; TMZ-containing therapies=TMZ alone and combined RT/TMZ.

In bold type were reported statistically significant results

*the sensitivity analysis was conducted only analyzing the studies with lower or lowest risk in selection bias, based on the results of the modified evaluation tool.
